# Use of domestic tools to downstage the reconstructive ladder in a patient with severe crush and degloving injury: a case report

**DOI:** 10.11604/pamj.2020.37.48.23998

**Published:** 2020-09-12

**Authors:** Amine El Harti, Komla Séna Amouzou, Mokako Lisenga Jacques, Mehdi Rizk Alaoui, Mounia Diouri

**Affiliations:** 1 Hassan II University, Ibn Rochd Teaching Hospital, Casablanca, Morocco,; 2 Department of Surgery, University of Lomé, Lomé, Togo

**Keywords:** Wound, trauma, plastic surgery, COVID-19, Africa

## Abstract

In the COVID-19's crisis, elective surgery and non-emergent cases were postponed; all other procedures have to be minimized. A 17-year male patient with severe crush and degloving injury over the thigh, gluteal, sacral, and perineum areas was admitted to our Department on the 16^th^ of March 2020. The patient presented soft tissue skin and muscle loss. A double Latissimus Dorsi and Anterolateral Thigh free flaps were indicated. However, due to the particular circumstance of the COVID-19 crisis, we applied domestic negative wound therapy (NPWT) using gauzes and wall suction. We obtained suitable granulation tissue after 17 consecutive days with this treatment. The raw area was then covered with an expanded split-thickness skin graft. The wound healed at 95%, and the patient was discharged on 25^th^ of April 2020. He was followed up in an outpatient setting with wound care and physiotherapy. This case showed that in a limited-resource setting, with available wall suction, the domestic NPWT is a versatile tool to promote granulation tissue.

## Introduction

When the World Health Organization (WHO) declared a global health emergency in January 2020 [1], healthcare systems worldwide were unprepared to deal with the COVID-19 pandemic. Although the WHO claimed that only 5% of infected patients might require critical care [1], the rationing of resources has been the standard adopted worldwide [2-4]. In Morocco, the number of cases was rising, as reported in data published by the WHO [5]; thus, the government declared a state of emergency on the 20^th^ of March. In the medical community, elective surgeries, and non-emergent cases were postponed. However, cases of severe trauma are time-consuming and require a certain amount of human and technical resources. The clinical presentation may guide through complex operative procedures. Exposed bones and tendons may require full vascularized coverage provided only by flaps to restore volume and function with a cosmetically acceptable result. In settings where flap surgery is not possible due to technical or environmental factors, trauma teams need to find out ways to minimize the procedure without adding on the morbidity to the patient. In our Department, we used in the past a negative domestic pressure wound therapy (NPWT) to convert limited cases needing flap coverage into skin graft coverage with good results [6]. We applied the same procedure to this more severe case of crush and degloving injury, because of the requirements of the moment. We report in this article the results achieved using this domestic NPWT to downplay the reconstructive ladder, as an example of our adaptability during the COVID-19 crisis and beyond.

## Patient and observation

**Figure 1:**
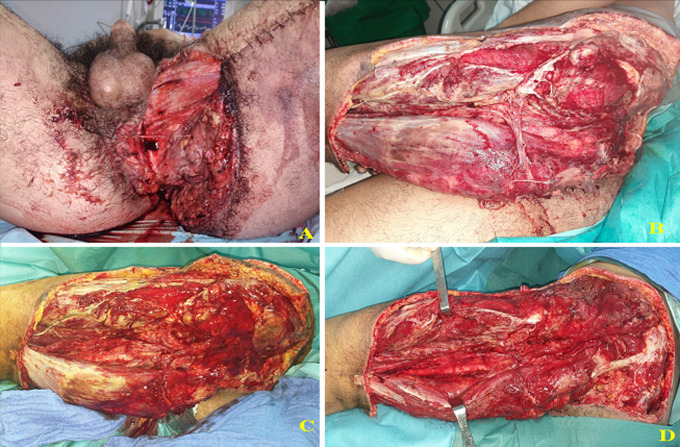
crush and degloving injury, presentation at admission (A), and after multiple debridement (B-D)

**Figure 2:**
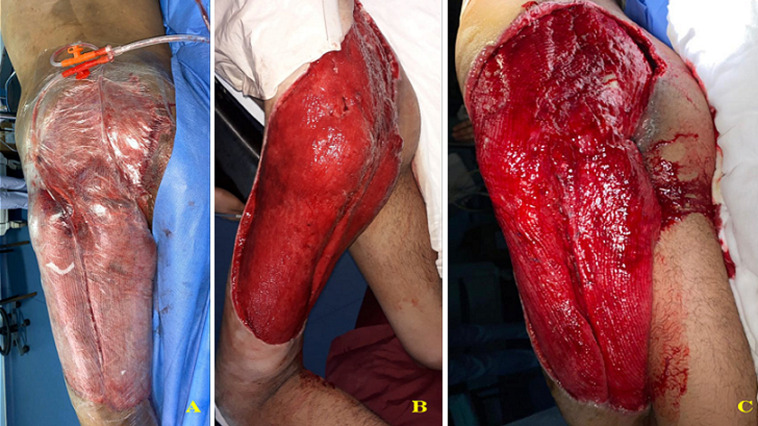
negative pressure wound therapy (NPWT), gauzes on the surface of the wound sealed with transparent adhesive dressings, a tube connects the wound to the wall suction; granulation tissue on the wound after 17 days of (NPWT) (B and C)

**Figure 3:**
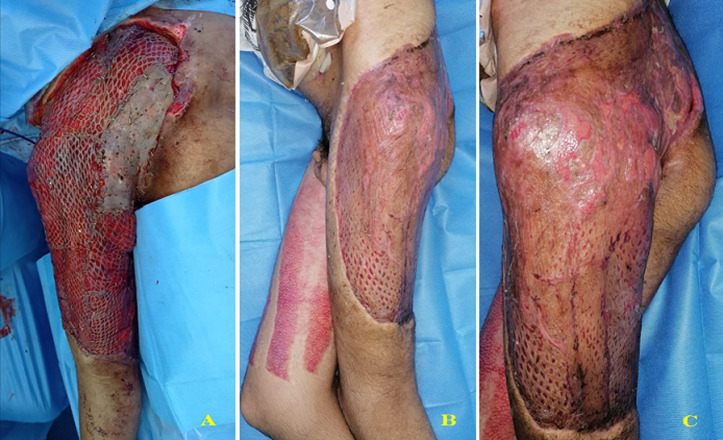
resurfacing of the wound with a split-thickness skin graft (A); near total wound healing 20 days after the skin graft (B and C)

A 17-year-old male patient sustained a road traffic accident (truck and bicycle collision) on the 16^th^ of March 2020. He presented with blunt abdominal trauma, a crush, and degloving injury to the left buttock, thigh, and perineal regions (Figure 1). We performed a diverting colostomy to prevent faeces contamination of the wound. Necrotic tissues, including skin and muscles as the Gluteus Maximus, the Gluteus Medius, the Tensor Fascia Lata, some muscles of the posterior compartment of the left thigh, and part of the Vastus Lateralis were all excised (Figure 2 A). Due to the extent of the defect associated with the sacral bone and coccyx exposition, a free latissimus dorsi (LD) and an anterolateral thigh (ALT) flap were indicated. Unfortunately, before we reached a local acceptable inflammation state for the reconstruction, we got into a state of emergency in which the government gave stay-at-home instruction. The medical staff was instructed to use medical resources efficiently. The anaesthesia team was busy dealing or planning critical care for COVID-19 patients. There was a limited room left for surgery of the magnitude of a double-free flap in our centre at this time. We decided to manage the patient according to our previous experience in using negative pressure wound therapy [6]. Multiple surgical debridements were necessary to remove all the necrotic tissues. Then, the wound was covered with gauze, sealed with transparent dressings, and connected to a wall suction under a pressure of 200 mmHg (Figure 2 B). We could manage to get a seal of around 90% using the tips described by Amouzou *et al*. [7]. The dressing was changed every three days under sedation. A granulation tissue appeared within 17 consecutive days of this therapy (Figure 2 C). The patient presented local colonization of the wound by an Imipenem resistant *Pseudomonas* spp. *Acinetobacter baumanii* sensitive only to the quinolone family; a split-thickness skin graft was performed to resurface all the raw areas in a one-stage surgery (Figure 3 A). On top of the graft, we applied the domestic NPWT again; however, with a lower suction pressure (150mmHg). An intravenous (IV) colicine and ciprofloxacin were given to the patient as an adjunct to the coverage surgery. The graft take was 95% (Figure 3 B, C). The patient was discharged on the 25^th^ of April 2020 (day 39). He was reviewed in the outpatient department every 4 to 5 days for wound care and physiotherapy. He was ambulating with a double walking cane. We acknowledged to him that some other procedures might be needed when the COVID-19´s crisis will be over should bulkier coverage still be required. The patient and his mother gave their consent to use the medical records for a scientific publication.

## Discussion

In this case, we have achieved an adequate wound healing for a complex defect that primarily required multiple complicated procedures. We moved from microsurgery down to skin graft in a span of 17 days. The serial debridement that preceded the use of the domestic NPWT has been conducted to remove all necrotic tissues, thus allowing granulation tissue to proliferate and avoid infection. Converting microsurgery into a low magnitude surgery as a skin graft is what we learned from this case. In China, where the COVID-19 has been reported for the first time, similar measures have been in use. For instance, in burn centres, surgeries were minimized, outpatient care was promoted, and telemedicine was widely used for the diagnosis and care of patients [8]. The goal was to take care of the patient as efficiently as possible while minimizing physical contact. In our case, our goal was to reduce the use of technical and human resources in a potentially resource-scarce environment where all attention is given to the COVID-19 at the expense of other cases such as the trauma cases. The reconstruction of the traumatic defect of the gluteal area is challenging and not well codified in the literature [9]. The indications are mostly based on traditional flaps used for resurfacing pressure injuries at this location. The use of free flaps, although exceptional in pressure injury settings, may be required for deep extended defects due to trauma or after excision of cancer [9,10]. The use of the Vacuum-Assisted Closure (VAC) therapy has been reported by Masami *et al*. [10] on a defect that was less extended than the one we treated in this patient. These authors achieved granulation tissue and coverage with a skin graft. However, in the long term, they were obliged to excise the skin graft and cover the defect with an ALT free flap as the wound was presenting with frequent ulcerations [10]. Frequently used flaps for such defects include the LD flap, the ALT flap, the scapular, used alone or in combination [9,10]. In our case, the commercial VAC was available, but the patient could not afford it. Therefore, our cheap NPWT was the best choice in this particular patient. The injury pattern guided the decision of the double flap. Although we obtained a wound healing that was acceptable for ambulation and physiotherapy, an unstable scar may occur, prompting the use of free flaps in the future, something the patient was acknowledged.

## Conclusion

The COVID-19 pandemic is giving us an unprecedented challenge in dealing with technical and human resources. The domestic negative pressure wound therapy is a simple and versatile tool in all settings with available wall suction. African medical teams are reputed to possess the capabilities to adapt to exceptionally limited resources to achieve great results. The adaptive strengths required during the current pandemic can be exploited even beyond the COVID-19´s crisis and enrich our decision-making process.
